# Supposed Demoniacal Possession

**Published:** 1850-04-01

**Authors:** 


### SUPPOSED DEMONIACAL POSSESSION.

*To the Editor of the "Journal of Psychological Medicine."*

Sir,—I take the liberty of forwarding to you a continuation and more minute detail of the dreadful sufferings experienced by me during many years, and which in your able quarterly publication you have classed under the title of "Supposed Demoniacal Possession:"—notwithstanding all your kind considerate reasoning, I still remain a most unwilling, yet strong advocate for the—in these enlightened times—generally unbelieved, unacknowledged notion of the occasional possession of mortals by evil spirits. My own unfortunate case has led me to this belief—to this acknowledgment—to this conviction. Happy should I have felt had I the power of supporting, as I had some years ago, a contrary argument and position : but with my own personally recognised affliction of demoniac possession, I cannot, with the slightest regard for veracity, otherwise than endeavour to maintain what, in the nineteenth century, may not perhaps inaptly be termed, newly-revived ancient opinions and doctrines.

And here, sir, will you allow me to express my great obligations to you, for having with generous urbanity listened to my narration, and notwithstanding your own were not in accordance with my views, yet permitting me the privilege of publication under your high authority. Be assured, sir, that such liberality of sentiment must inevitably tend to the promotion of the true interests and development of knowledge.

In returning to my subject, I must premise that I shall have to tax largely the faith, or, as others may say, the credulity of many : but confident of the truth of that which I write, it is without hesitation that I boldly enter into the arena of the press, prepared to maintain the present existence of evil spirits, and to set forth their influence on the sons of men.

It was, as I observed in my last essay, as I was taking a quiet walk one evening about six years ago, in the outskirts of the metropolis, that I suddenly experienced the sensations of the sound of apparently human voices. There was no human being on the spot, but as the sounds were those of conversation between one person and another or others, I endeavoured to trace their origin: such attempt was ineffectual. A boy came within sight, amusing himself in some ruinous buildings at a distance,—the sounds appeared to proceed from him. I called and ran to him, but being alarmed he speedily made his exit. The language then appeared to emanate from some fowls ; I knew these birds incapable of producing such sounds, and entertained myself by thinking on the marvellous stories in the "Arabian Nights," and in our Indian transmigratory tales, and then reasoned that persons in a cottage not far from the fowls might possibly, by ventriloquism, have deceived me. I approached the cottage, and watched it a considerable time; the voices then seemed to be those of persons in the interior of it, but no one appeared at the windows, and some labourers passing by I walked away. The voices then evidently came from these men, gradually becoming more and more indistinct, until I lost them momentarily. The words used were, "Who is he?—What is he?—Do you know who he is?" The responses were, "He is the Devil's own—He is Satan's own." Shouts of laughter and derision and questions of doubt afterwards succeeded, and then as I walked with rapid pace through London and its environs, observations were made that I was walking for a wager—that I was the man condemned to live hundreds of years, and to walk the earth—that I must be Satan himself—that no human being could possibly walk so fast, without rest and refreshment and so great a distance—that I had boots on which no doubt concealed my cloven feet—as for my tail that was hid under my frock coat. Alternately I felt annoyed and amused, proud of my exploits, and then grieved at them—smiling occasionally on recalling to recollection the verses of "The Devil's Walk," and comforting myself that with daylight I should be recognised, and be once more myself again. But I was unfortunately doomed to disappointment; the voices still pursuing me apparently from the various passers by. At last the uproar, din, expressions of revenge and maledictions became so vehement, that I determined on going to an hotel where I was well known; the moment I thought of which the voices exclaimed, they would soon find out who I was, and would not leave me until they did; and a boy who kept pace with me in the road or on the path, I now imagine for amusement, appeared to denounce me as a criminal to every one. He then preceded me, and I missed him near the hotel, and thought he had slipped in there; but as I was afraid of nothing that I had done, I walked in, and asked the waiters if a boy had just been there to inquire about me, and whether he had said anything and what against me? but they assured me they had not seen him; and I desired if he came that he should be shown in to me, that the proprietor might satisfy him as to who I was, and that I was incapable of acting as he, and the others who had commissioned him, had said or thought. No boy, however, made his appearance; and after a short stay, and partaking of some refreshment, I ventured out, as I hoped quite released from the tortures of the previous twelve or fourteen hours. I crossed Waterloo Bridge, intending to visit a friend who was then in the State House, and whose liberation I was anxious to effect. Whilst on the bridge a voice sounded as if from the water, exclaiming, "There he is again!—there he is!—that is the man!—he shall not escape!"—I looked to the water and perceived several boats, and thought that possibly the boy was crossing by the ferry, but I

could not discover him. On arriving on the Surrey side of the river, many were the voices uproused against me, clamorously insisting on my punishment; that of hanging was suggested; far and near they pealed on my ear, until when near the gates of the Bench, I actually was fearful that I should not be allowed to enter, in consequence of the multitude, which I could not see, but whose clashing of weapons of every description and cries and threats of revenge I could distinctly hear. At first I thought, from not seeing them, that my accusers had obtained access within the walls; but, when I entered, not observing any extraordinary number of persons, I considered they were behind me, and congratulated myself on having arrived in a place of safety when I got into my friend's sitting-room. Communicating the circumstances to my friend, he only stared with surprise at me, and said I need not be under the least apprehension of anything whilst with him. The noise and din and clashing of arms and martial music appeared then to increase, and calls were made for the governor to give me up to the infuriated mob, when I offered to prove to his and their satisfaction, that my conduct through life had not been so bad as they represented, to which proposal they assented; and the voices of some of my accusers then appeared to sound within the walls, and the governor seemed to be appointed judge. Several voices then made accusations against me; the window of my friend's apartment was open, and I stood at it, admitting some of the charges and palliating or denying others; the voices, very singularly, resembled the sounds of those with whom I had had transactions; and I heard distinctly the assumed voices of my relatives and friends. I was evidently put on a regular trial, and witnesses on both sides spoke against and for me. The voice of one was clearly that of a being of authority. I thought at first it was the voice of the governor, but I have since known differently, as it has ever since remained with me. It is a strong powerful voice, such as, to speak at once intelligibly; Satan himself, or one of his chief emissaries, might be the possessor of. For some time, however, I was inclined to believe it to be the voice of the Angel Gabriel, or that of some high angelic spirit; but when I subsequently heard it associated with the dreadful imprecations and language used by other voices, my opinion altered. My heart and brain seemed at the trial to be open to the view of every one; and I replied to the charges by my thoughts, without utterance. The evidence was directed to be written down, and the following day I went again at the time appointed, prepared for another examination; in the interim the voices continued with me, and I wandered about the entire night in much the same manner as I had the previous night. The next day I stood at the window of my friend's room as before, and the proceedings of the trial were commenced; but little however occurred, as the parties appeared satisfied with my mental proposition of compensating, to the fullest extent in my power, all persons that I had injured in thought, word or deed. Some of the voices which seemed to belong to a band of musicians, who had performed martial airs both on that and on the previous day, which had sounded as if played outside the walls, demanded money, and said, I think, they were twelve in number, and wanted £3, ultimately agreed to take £2, which I told my friend, and that I must get rid of them at any sum; and begged him to send for a messenger to whom I could give the money for them—which to appease me he did—and I handed the man the £2 to take to the band waiting outside the walls or at the gates. These £2, however, my friend very wisely got from the messenger immediately again, under the pretence of shutting the door after him, and afterwards the same day transferred to my mother. I had asked my friend previously, both days, if he had heard the voices and noises, but he declared he had not. In fact, to say the truth, I did once or twice think that probably my friend might have mesmerised me, for I could not account for the sounds in any natural manner; but I soon divested myself of this impression, when the thought arose that I heard the voices before I called on him on the previous day. I could not, even during the trial, although it was so solemnly conducted, and the consequences impending were serious to me, frequently avoid smiling at the ridiculous impossibility of the affair, as I was in a room, could not see my accusers, knew that I ought to be tried in the customary way, by a judge and jury, if I had done any criminal act, and in a law court, and yet I could not distrust the evidence of my own ears. I tried to pray, and then laid down to compose myself. My friend had very kindly on the previous day written to my mother, describing my excited state, and she arrived, and I returned with her home; but even in the cabriolet "The Devil's own" resounded from some voice that kept pace with us; and I could not help imagining that some one had placarded the back of the vehicle with that dreadful name, and I made the driver alight to see if anything or any one was at the back of the cabriolet; and when we got out, I myself went to be satisfied. At my instance we did not return to my domicile until late in the evening, as I thought the writing would not then be legible, and the reiteration of the words would cease. No such good fortune attended me; and, as I mentioned in my last essay, I afterwards experienced the sounds of about seven different voices—two those of females, which number continued with me many months; afterwards four remained with me nearly two years; and since then two, a male and female, have been my constant companions. Last September they nearly left me, and occasionally they are very quiet, so that the voices nearly resemble a humming noise, as though at a distance; but at other times I hear them as distinctly as ever, and sometimes, if I listen attentively, I can hear their discourse; and the voice of the male is, as I before named, that of the spirit in authority. I trust to be excused for entering thus explicitly and fully into details of the commencement of my affliction, which has now been of six years and four months' duration; but I thought such might be interesting to the philosophic and contemplative mind.

Whilst at home I was incessantly tormented by the evil spirits by day—the idea can be but inadequately conceived, of seven persons continually talking to each other, or to me, or of me, on all kinds of subjects, making observations, satiric or pleasing, on what I thought, or did, or said, or intended; carrying my attention from the subject on which I might have been engaged, into other extraneous channels, thereby preventing my reading or writing, thinking, acting, or speaking, without confusion or forgetfulness; incapacitating me for the exercise of my profession, which I had increased to a very large extent, and which I have, chiefly on this account, been obliged to relinquish; and by night causing me, by the most horrible proposals, threats, and artifices, to keep awake for five or six, or more hours, every night, in the most dreadful state of agony that it is possible for the human mind to portray. Several of the spirits seemed more violent than the others,—they proposed all kinds of indignities to God, and to our Saviour, and to the Holy Spirit; they uttered the most horrible language, curses, oaths, and imprecations, and I have experienced the utmost difficulty in preventing myself using the same, having been in the hourly habit of hearing these fiendish sounds for so many years; but thanks be to God! I have never made use of an oath or an improper word in my life. The two or three or four hours' rest that I had in the morning, barely sufficed to eke out my wretched existence for the day.

Amongst the proposals made by the evil spirits to me, were primarily, that I should worship Satan, or acknowledge him as my superior, or give myself up to him, or trample on the cross of Christ, or disown the supremacy of God. On doing, or thinking any such things, unbounded power and wealth should be mine; I should gain whatever I required, and do whatever I wished; I should be able to communicate with all persons, however distant they might be; I should have power to influence them as I pleased, and to see what they were doing, and where they were, and who were with them, and to dive into their thoughts and actions, and to know and see generally both present and future whatever I chose; and lastly, all favours I desired should be granted me by the fair sex.

And here I may mention, that on mentally addressing any one, if I wished to arouse his attention, his name was to be repeated, mentally, three times; if he belonged to the fraternity he would reply, "well," or "what do you want," or "Oh, you are one of us, are you?" and if I had not before addressed him he would appeal to another, or superior spirit, who would reply in the affirmative, and mention my name, and in a moment an answer would be given to any question propounded.

The evil spirits pointed out the various incalculable advantages of all these powers, and directed me how to exercise them; but by the grace of God, through the merits of my Saviour, I have been enabled to withstand the temptations of wealth and power by never once in thought or deed acknowledging the influence of Satan. In some respects, however, I have unintentionally fallen; and now I

must, as I before said, tax the faith or the credulity of many in asserting that one of the female spirits, who usually took my part, and behaved with greater kindness to me than the rest, having informed me that she was a female whom I had seen about three or four times, and admired when I was about fifteen years old, I gradually fell under her influence, and as, after the clear exposition of my heart and brain at the trial, I had nothing to disguise, I repeatedly allowed her hand apparently to pass, as I seemed to feel it, through the various divisions of my brain, and also through my heart.

Blessed with a contented disposition, although very humble is my lot now, Satan, with his power and wealth, I have been enabled to resist; as regards the other powers, it has happened that I could, and have, sometimes exercised them, but I have placed them to the account of sympathetic feeling; and it is singular that if at any time I wished particularly to meet or see a person not living at a distance, I have almost invariably met or seen him. For some length of time I have had a difficulty in divesting myself of the notion that I could communicate with any one at a distance, and see and know anything I pleased anywhere, both present and future, which may really be termed clairvoyance. The last power, before mentioned, I almost thought I had, but this might be classed with sympathy of feeling, or animal magnetism. On all these subjects I am, however, still sceptical, although I have certainly occasionally met with rather startling and surprising incidents. It ought to be noticed that frequently, in thinking, writing, reading, or shaving, I am apt to become lethargic, or in a comatose state. My sense of touch is peculiarly delicate, and all my other senses are particularly fine. It is one of the regrets that I entertain, that I did not keep a diary of some of the various conversations that occurred between the evil spirits themselves and those with me; but had I done so, a large folio volume would hardly have sufficed for the long period of upwards of six years to which my sufferings have been extended. I am not addicted to falsehood, and I can pledge myself that all that I have written on this subject is perfect truth.

I feel, and am fully aware, of the difficulty in which I am placed in detailing feelings that to the general world will appear, and perhaps be pronounced, to be hallucinations; but we have yet to learn that there are not evil spirits still existing and allowed to roam at large on the face of the globe. Our Saviour, as every one is acquainted, did not eradicate them during his sojourn upon earth, nor is there any account of their total extinction during the time of the Apostles; indeed, as they continued after our blessed Saviour's ascension, it is far from anything like probability that the subordinate power of the Apostles should drive them away,—then, doubtless, evil spirits still are permitted to speak to and influence mortals. I have always enjoyed the entire possession of my senses; I have never seen any of the spirits; I did not believe in them until this visitation,—but voices occurring in this manner cannot be those of the human race. I can readily imagine the recurrence of the brain to vocal or instrumental music contrary to one's own wishes, —this would be a species of reverberation of the sound of that which one had previously heard; again, the recurrence of a murderer to the scene of his guilty catastrophe, is attributable to his thoughts constantly dwelling on the subject of his crime; but voices speaking distinctly to one on topics unthought of at the time, using expressions to which one has been unaccustomed, pointing out what one has wholly forgotten or omitted, or done or thought wrongly or amiss, cannot be caused by reverberation of sound or recollection of ideas. They are not the reiteration of sounds, the refraction on the mind of bye-gone objects; here the brain, it must be borne in memory, is not approached through any other but *one* sense, and that is the faculty of hearing. Whence can sounds, then, so dissimilar, so dissonant, to one's innate nature originate? How can one shudder and shrink, on the utterance of hellish sounds as from a by-stander, if such were not independent of oneself—had no corresponding relation in one's heart or brain,—nothing sympathetic, to induce or to allure them? No concurrent thought or wish has been lurking there, to produce such dreadful impiety. It is, it has been, only through the ear that such voices have reached my brain, —by the aid of Divine Providence they have never affected my heart. On reflection and re-reflection, with my little stock of information, I cannot in any other manner account for these voices, than that they emanate from Satan. I have been highly favoured in having been naturally of a religious frame of disposition, and for some years, singularly, kept a written daily journal of all my sins of thought, word, deed, and omission; and I was in the daily habit of reading the Scriptures and religious works. I remember having been much pleased to discover, as I thought, the meaning of the passage relating to the apple of Adam and Eve; the apple I merely considered allegorical, and that the concupiscence, or excess of passions of our first parents, when not confined within the bounds of reason, really formed the groundwork of their fall. Since my affliction, however, I have thought that Satan, allegorically under the type of the serpent, did actually *speak* to them, and did also in like manner *speak* to our Saviour; indeed, on reference to the various passages throughout the Holy Bible, it will be found that the version of sound that I now entertain and support is the true and chief mode by which, independently of our own personal evil thoughts and desires, Satan is permitted to hold intercourse with man. And, I have no doubt, the more this matter is reasoned on and considered, the firmer will be the conviction at which I strive.

With this knowledge, that our adversary the Devil is going about like a roaring lion seeking whom he may devour, whom we should resist, steadfast in the faith; let us turn our thoughts from things below to things above, and by penitence, faith, and prayer, avert or render harmless so dire a calamity as that of demoniacal possession, and which we shall be enabled to effect only by the grace of God, through the mediation and merits of our Saviour, and the influence of the Holy Spirit.—I beg to remain, &c. R. F.

